# Cloned Defective Interfering Influenza RNA and a Possible Pan-Specific Treatment of Respiratory Virus Diseases

**DOI:** 10.3390/v7072796

**Published:** 2015-07-08

**Authors:** Nigel J. Dimmock, Andrew J. Easton

**Affiliations:** School of Life Sciences, University of Warwick, Coventry CV4-7AL, UK; E-Mail: a.j.easton@warwick.ac.uk

**Keywords:** defective interfering RNA, influenza virus, respiratory disease, treatment, therapy, prophylaxis, antivirals

## Abstract

Defective interfering (DI) genomes are characterised by their ability to interfere with the replication of the virus from which they were derived, and other genetically compatible viruses. DI genomes are synthesized by nearly all known viruses and represent a vast natural reservoir of antivirals that can potentially be exploited for use in the clinic. This review describes the application of DI virus to protect from virus-associated diseases *in vivo* using as an example a highly active cloned influenza A DI genome and virus that protects broadly in preclinical trials against different subtypes of influenza A and against non-influenza A respiratory viruses. This influenza A-derived DI genome protects by two totally different mechanisms: molecular interference with influenza A replication and by stimulating innate immunity that acts against non-influenza A viruses. The review considers what is needed to develop DI genomes to the point of entry into clinical trials.

## 1. Introduction

In very early studies of influenza virus replication in embryonated chicken’s eggs von Magnus described how, during successive undiluted passages, the yield of infectious virus cycled from high to low. Further analysis suggested that during the periods of low virus infectivity most of the virus particles present were “incomplete” and unable to initiate a productive infection [1]. It was not until analysis of virus genomes was possible that it became clear that the defect in these non-infectious virus particles resulted from large (up to 90%) deletions in one or more of the eight viral genome segments [2,3,4,5,6,7]. In the presence of these defective particles the production of infectious virus is severely diminished and this led to the designation of the mutants as defective interfering (DI) viruses [8]. The production of DI viruses has been demonstrated in almost every virus system studied. Many DI viruses contain deletions so extensive that they do not encode any functional protein. The deletions arise due to errors in replication in which the polymerase carrying out the replication process leaves the template nucleic acid, and either rejoins at a distant point on the same or another template or, in the case of some DI viruses with RNA genomes, switches from the template to copy back along the newly synthesised RNA. In viruses with segmented genomes such as influenza virus, DI RNA can arise from any genome segment and the packaging of this with the other full-length segments results in a DI virus particle. Most natural DI influenza virus preparations contain many different DI genomes. All influenza DI RNAs have a central deletion leaving the termini of the RNA intact. These carry the replication and packing signals essential for the DI RNA to be propagated and packaged [3,4,9]. Deleted RNAs can arise from any of the genome segments, but DI RNAs derived from the largest segments 1, 2, and 3 are the most frequently observed either *in vitro* [4,9,10] or *in vivo* [11]. Within a segment many different break points have been observed, so that many different DI RNA sequences can arise from just a single segment. Subsequent deletions can also arise. In a preparation of influenza A DI viruses more than 50 different DI RNAs were detected [10].

DI viruses are defective because the deleted genome lacks an essential gene required for replication. In order to replicate, DI viruses require assistance of the infectious virus from which they were derived, or a genetically compatible related virus, to provide the missing gene products. This is referred to as a helper virus. DI virus production is optimal in the presence of a large amount of helper virus but as the DI virus replicates it reduces the yield of infectious helper virus. The reduction in helper virus arises because the smaller DI genome is replicated much faster than the larger parental genome, so that more DI genomes are synthesised in unit time until the DI genome predominates. This provides two advantages for the DI genome; stochastically DI genomes are then able to compete more effectively for essential product(s) synthesised in limited amounts by the infectious helper virus or the host cell, and secondly the abundant DI genomes are more likely to be packaged into new virus particles. Because of the dependence of the DI virus on helper virus to provide the essential proteins DI and helper virus particles are structurally identical. It is important to appreciate that not all viruses with defective genomes are able to interfere efficiently with the replication of their helper virus.

The detection of DI viruses *in vivo* is technically difficult as they appear to be generated in only low levels, but DI/defective genomes have recently been reported from infections of a number of vertebrate species including humans [2]. While it is possible that DI viruses are simply the result of errors of replication and have no evolutionary significance there has long been speculation about a role for DI viruses in natural infections. One possibility is that DI viruses are evolutionarily important and in natural infections restrict the extent of damage caused to the host while allowing infectious virus to be produced. The host survives the infection and the virus is able to be disseminated to new susceptible hosts. This argument suggests that an infection starts at a low multiplicity of infection. Along with new infectious progeny, DI genomes and viruses are produced and infect the surrounding cells. High levels of infectious virus permit coinfection of cells with a DI virus, and with the resulting propagation of the DI virus, and its introduction into yet more susceptible cells. As the ratio of DI virus to infectious virus increases due to the replicative advantage of the DI genome the amount of infectious virus is reduced, allowing the host time to mount immune responses and defend itself. In addition, or alternatively, DI viruses have been found to stimulate innate immune responses which both act against the infection and potentiate anti-viral adaptive immunity [2,12].

The ability of some DI viruses to severely inhibit replication of the helper virus *in vitro* led to the suggestion that these may be applicable as natural antivirals with clinical applications. While some early *in vivo* studies provided hopeful results, much of the data were poor, sketchy or unreproducible [5]. This led to the almost complete abandonment of the study of DI viruses as antivirals until recent technical developments reopened this area as a promising avenue for exploration. One of the most significant problems was that naturally produced DI virus preparations consist of a complex mixture of DI genomes which is difficult to generate reproducibly; further there was the possibility that each DI virus in the population had a different capacity to interfere with helper virus replication. This has been solved by using molecular cloning techniques to produce homogeneous populations of DI viruses with a single DI genome sequence with reproducible characteristics that is stable on passage [13,14]. The first outcome of this was an appreciation that while all DI viruses interfere with helper virus replication *in vitro* not all DI viruses are able to protect from infection *in vivo*, as described below for Semliki Forest virus DI viruses [13]. Over recent years, significant progress has been made with a DI virus derived from an influenza A virus (IAV), which has been shown to be able to both therapeutically and prophylactically protect experimental animals from lethal challenge with influenza and other respiratory viruses.

The influenza A DI 244 virus was generated using recombinant virus technology to molecularly clone a single DI RNA that arose spontaneously [14]. DI 244 RNA is 395 nucleotides in length and was derived from segment 1 (2341 nucleotides) of influenza A/Puerto Rico/8/34 (PR8; H1N1). The DI 244 RNA contains 244 nucleotides from the 3′ end and 151 nucleotides from the 5′ end of segment 1. This DI RNA was recovered from cDNA into a DI virus using molecularly cloned infectious influenza A PR8 as helper virus. As described below DI 244 virus protects mice and ferrets from disease caused by a number of different IAVs. DI 244 virus also protects from disease caused by the genetically distinct influenza B virus and the paramyxovirus pneumonia virus of mice. The data indicate that DI 244 in particular and DI viruses in general have the capacity to beneficially modulate virus infection. This provides a new avenue for exploration of a novel class of antiviral agents for the treatment of clinically important virus infections. In this review we will consider the features that are important in the application and development of DI viruses as antiviral agents with the focus on DI viruses from viruses with RNA genomes, although many of the principles will also apply to DNA-based DI viruses.

## 2. Delivery Vehicles for Influenza DI RNA for Treatment *in Vivo*

As with any other potentially therapeutic RNA, a key question is how best to deliver the DI RNA *in vivo*. The problem is both targeting delivery as accurately as possible to the desired recipient cells, and protecting the single stranded DI RNA from degradation by ubiquitous ribonucleases. While delivery of therapeutic RNAs to cells is usually inefficient, viruses have evolved highly specific mechanisms to introduce their nucleic acid into target host cells and this offers an effective means of delivery of a nucleic acid provided that it can be packaged into a virus particle. For an influenza DI RNA there is no problem as IAV provides an ideal delivery vehicle. Administration via the respiratory tract ensures that DI RNA is delivered only to cells, which carry influenza virus receptors and can be potentially infected by an incoming infectious influenza virus. In this way DI RNA is delivered only to cells that are potential virus targets. At the same time packaging into a virus particle protects DI RNA from ribonucleases.

Cells transfected with plasmids expressing the DI 244 RNA and all of the RNAs required to generate infectious influenza virus make nascent virus particles that spontaneously package the DI RNA. A critical requirement is to avoid an excess of DI plasmid as this is highly interfering and results in a very low virus yield [14]. During the genome packaging process an influenza virus particle packages only a single copy of each of the eight genome RNA segments and, if a DI RNA is present, it competes for packaging with its cognate full-length segment [2]. It follows that a particle that contains DI RNA is *ipso facto* non-infectious. A necessary consequence of the process is that some infectious helper virus is also formed, and this is essential for further amplification of the DI virus. DI and infectious helper particles are almost identical as the latter provides the proteins that constitute the DI virus. They differ only in the replacement of full-length segment by the DI segment so that downstream processing technologies already in use for infectious virus can be used for DI virus.

In an influenza DI virus preparation the ratio of DI particles: infectious particles can be in excess of 100:1. Infectivity has to be removed before the DI virus can be used to treat an infection. It has not been possible to separate DI influenza viruses from the infectious helper virus using physical procedures as the size difference between the DI RNA and the cognate genome RNA segment it replaces does not generate any significant difference in attributes such as particle size or density. This is the case even for DI viruses such as DI 244 where the 2341 nucleotide genome segment 1 is replaced by a 395 nucleotide DI RNA. An effective alternative that requires no physical separation is UV-irradiation. UV irradiation preferentially targets nucleic acid molecules, with the frequency of destructive events determined by the length of the target molecule, *i.e.*, larger nucleic acids receive more “hits” in unit time than smaller ones. In principle a single hit on the infectious genome is sufficient to abolish infectivity. The difference in size between the DI RNA (typically only a few hundred nucleotides) and the infectious genome (approximately 13,400 nucleotides) [14,15] is sufficient to ensure that infectivity is lost at a rate that is enormously greater than the loss of protective capacity from the small DI RNA and the differential rate is sufficient for this process to be effective.

One theoretical concern is that if any full-length segment survives UV irradiation, it might end up in the same cell as a natural infectious IAV and form a recombinant. The influenza viruses all have segmented genomes, and recombinants can be formed by exchange of segments (genetic reassortment) between two or more viruses (but only IAV with IAV, and influenza B with influenza B) infecting the same cell. For DI 244 virus this could only occur with IAVs. During its preparation DI 244 virus is UV irradiated to destroy helper virus infectivity, while leaving the short DI 244 RNA intact. This means that many of the full-length segments will have been destroyed and will not be available for any genetic interaction making such an exchange considerably less likely than might occur during a coinfection with two fully infectious IAVs. When a cell containing DI 244 virus is infected with another IAV, as would happen during natural infection, the incoming virus will act as a helper virus and will replicate and package the DI 244 genome. The DI 244 RNA will, therefore, be packaged into the particle produced by the challenge virus, and not the original PR8 strain. Due to the nature of the genome packaging process DI 244 RNA replaces segment 1 of the infecting virus. In a formal sense this is a reassortant virus. It is this process that ensures that the levels of DI 244 RNA are amplified during an IAV infection and that the DI 244 RNA is able to suppress the natural virus replication to a level below that generates signs and symptoms of disease. The new DI 244 virus particle constituted with components of the infecting virus is, as with all DI viruses, unable to replicate autonomously. Because the immune system responds to this virus particle it eliminates both the natural infectious virus and the amplified DI 244 RNA in the normal way. We have shown in mice and ferrets that the level of DI 244 RNA is reduced and eliminated within the same time frame as the infectious virus genome RNA.

Finally, we attempted in the laboratory to find reassortants, which have acquired the full length HA gene from a DI 244 virus preparation. In these experiments we coinfected mice with DI 244 virus and with an infectious virus with an antigenically distinct HA. We used an exceptionally high dose of infectious IAV (2 × 10^4^ LD_50_ or approximately 2 × 10^7^ IU) to provide optimum conditions for generating a reassortant virus. This infectious dose was far above any that might be naturally encountered in humans. Potential reassortant viruses were identified by positive selection with HA-specific monoclonal antibody under conditions, which favour such reassortment. In two experiments a single potential reassortant was found, but this could not be discriminated from possible residual helper virus present in the very large inoculum.

Despite much study there is no evidence for homologous recombination with any non-IAV with this type of genome, and no recombination has been detected even between closely related viruses in the same taxonomic family. There is certainly no risk of homologous or any other form of recombination with viruses of a different taxonomic family. The underlying principles of the elimination of DI 244 RNA from the respiratory tract with amplification only in the presence of an infectious IAV, the restrictions on recombination/reassortment of negative sense RNA viruses together mean that there is no practical likelihood of generating a recombinant virus.

It would be ideal if DI virus could be made without the need for infectious helper virus and subsequent UV treatment. In principle, this could be achieved if the missing viral function was provided *in trans* by host cells that had been engineered to express the required protein. However, some virus proteins such as PB2, the protein encoded by segment 1 that is replaced by 244 DI RNA in a DI virion and which binds to the 5′ cap structure of eukaryotic mRNAs, are likely to be cytotoxic [16]. Nonetheless, such toxicity is likely to be a property of the individual protein as a helper cell line expressing the paramyxovirus phosphoprotein, a constituent of the viral polymerase, has been described [17]. Low virus yields are a separate problem and in this example the helper cell yield was nearly 1000-fold lower than that achieved by a wild-type virus infection.

It is generally held that that influenza particles package one of each of the eight viral RNA segments, regardless of their length [18,19], so it is possible (but not known) that a single virus particle could carry more than one DI RNA. IAVs form a homogenous genetic group, which can exchange RNA segments by RNA reassortment whenever two different IAVs infect the same cell. Such genetic interactions are generally promiscuous, although there are some preferences as to the combinations of the 8 RNA segments [20]. One of these is the pairing of HA and NA segments, which have to balance the avidity of the expressed HA for the N-acetyl neuraminic acid (NANA) cell receptor with the ability of the expressed NA enzyme to cleave the NANA. The 244 DI RNA has been packaged by a number of different IAVs including the human strains A/PR/8/34(H1N1) and A/WSN (H1N1), and the avian strain A/mallard/England/7277/06 (H2N3), demonstrating the conservation of replication and packaging signals between different IAVs [14].

The influenza A strain selected as delivery vehicle for 244 DI RNA in all the preclinical studies performed to date has been A/PR/8/34. Isolated in 1934, there is little herd immunity to A/PR/8/34 in the human population, as people who might have experienced infection are now over 80 years old, and what immunity they had will have waned over the years. Laboratory strains of A/PR/8/34 are clinically avirulent, and even with high doses failed to infect human volunteers [21]. Should immunity to A/PR/8/34 appear in the human population in the future, 244 DI RNA could in principle be packaged using reverse genetics with the envelope proteins of any suitable IAV.

To get protection that involves amplification of the 244 DI RNA by an IAV, it is necessary for the DI and infectious viruses to enter the same cell, so both should use the same cell receptor. This is maximized by delivering the DI virus intranasally to the upper respiratory tract so that the DI and the naturally circulating viruses will arrive at the same anatomical location and infect the same cells. Avian or egg-adapted strains of influenza virus preferentially bind to alpha2,3 NANA receptors while human strains use alpha2,6 NANA receptors. Cells bearing these receptors are concentrated in different parts of the respiratory tract, so that a DI virus that uses alpha2,3 NANA receptors and is inoculated into a mammalian host is unlikely to efficiently protect against an infecting virus that uses alpha2,6 NANA receptors. This was borne out experimentally in ferrets (unpublished data). Examination of the binding capacity of different A/PR/8/34 viruses identified one that bound to approximately the same extent to each type of receptor and thus could protect against an infecting virus that uses either an alpha2,6 NANA or alpha2,3 NANA receptor [22]. However, once DI RNA is replicated by a naturally circulating influenza strain, the newly synthesized DI RNA molecules are packaged by the proteins synthesized by that naturally circulating virus into new DI virus. Thus all *de novo* progeny DI virus has the same receptor preference as the naturally circulating virus. The newly synthesized DI virus then disseminates throughout the respiratory tract and protects an ever-increasing number of susceptible respiratory tract cells.

Cell specificity is less of a problem where protection by DI IAV is aimed at non-influenza A respiratory viruses as these do not amplify this DI RNA, and protection depends on stimulating soluble mediators of innate immunity like interferon type I. Such mediators can diffuse to adjacent cells of the respiratory tract and, as a result, the treatment is largely independent of virus receptor specificity.

## 3. Reciprocity in the Amounts of DI Virus and Infectious Virus in the Effective Treatment of the Mouse

**Figure 1:**
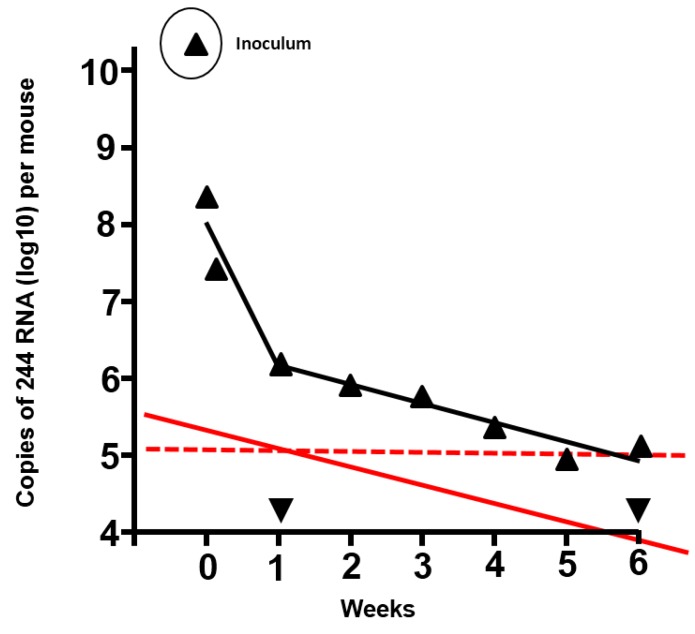
Duration of residence of 244 RNA in the lungs of mice inoculated with 12 µg 244 DI virus protein, and prediction of the duration of protection from infectious challenge virus. The amount of DI RNA was determined by quantitative PCR (▲) (adapted from [2]). ▼, PCR signal from lungs of mice that were inoculated with diluent. Mice treated with 1.2 µg 244 DI virus were protected from lethal challenge with infectious IAV after 7 days had elapsed. However, 244 DI virus did not protect at 14 days after treatment, indicating that the DI RNA had declined to a sub-protective level and that no anti-DI virus immunity had been generated. The solid red line shows the amount and decline of the DI RNA in the lower DI virus dose, assuming that the amount of DI RNA in the lung and the kinetics of decline were proportional to the higher dose (in black). The red dashed line is an extrapolation from the 1.2 µg dose that was still protective after 7 days and suggests that the 12 µg dose of 244 DI virus would still be protective at 5 to 6 weeks after treatment. It is calculated that DI RNA from the high dose declines to zero in approximately 6 months.

One of the key messages to emerge from the recent studies of influenza DI viruses as antivirals is that their efficacy is determined by a quantitative relationship with the infecting virus, and this applies equally to their inhibition of IAVs by interfering with replication and packaging and of non-influenza A respiratory viruses by stimulation of interferon type 1. An important aspect of this relationship is that the number of infectious particles inoculated, and not the infectious or lethal dose determined in the animal system in question, is the important factor, as the latter can vary from virus-to-virus by many orders of magnitude. Table 1 shows data for an IAV system, where the ratios of DI virus: infectious virus have been titrated in 10-fold steps. Protection of mice with a fixed amount of DI virus diminishes as the amount of infectious challenge virus increases, until the infectious virus effectively swamps the action of the DI virus, and protection is abrogated. A reciprocal phenomenon occurs when the infectious virus dose is fixed and the DI virus dose is increased. For 244 DI virus the lowest ratio to offer protection was provided by the application of 3400 DI particles: 1 infectious virus particle. However, it is likely that lower amounts of both components enter cells of the respiratory tract (see, for example, Figure 1 where the amount of DI virus drops precipitously soon after inoculation). Thus the use of an infectious inoculum that contained a critical lethal mouse dose (LD_50_) but which had a very high infectious particle: LD_50_ ratio may explain why, in the past, DI virus-mediated protection failed [23]. The infectious dose of viruses acquired by the natural route is generally very low and would provide a highly favourable DI particle: infectious virus particle ratio. For example, the airborne influenza A human infectious dose is 0.6 to 3 infectious units [24]. This bodes well for the efficacy of DI virus treatment in the clinic.

**Table 1:** Ratios of influenza 244 DI virus particles (DIP): particles of infectious virus (VP), and the protection of mice from challenge with infectious influenza A/WSN virus.

		A/WSN VPs *												
			10^5^			10^6^			10^7^			10^8^		
			P	DIP:VP		P	DIP:VP		P	DIP:VP		P	DIP:VP	
244 DIPs *	3.4 × 10^10^		++++	340,000		++++	34,000		++	3400		±	340	
	3.4 × 10^9^		++++	34,000											
	3.4 × 10^8^		+++	3400											
	3.4 × 10^7^		+	340											

* 244 DI virus and infectious IAV (A/WSN) were mixed and inoculated intranasally under light anaesthesia. DI RNA was estimated by quantitative RT-PCR and DIP numbers assume that there was 1 DI RNA molecule per DI particle; the WSN VP number assumed that there were 100 particles per 50% egg infectious dose. Mice were assessed clinically and weighed each day; ++++, denotes complete protection with no clinical signs and no weight loss; +++, transient mild illness and slight weight loss with rapid recovery; +, all mice become ill, but approximately half recovered; ±, illness and death were delayed by 3–4 days, and all died. Note that without added DI virus all infected mice died in 4–6 days. P, protection; DIP, defective interfering particles; VP, particles of infectious A/WSN; green, denotes protection; red, denotes no protection. Adapted from [2].

## 4. Influenza DI Virus and Immunity

One of the possible limitations imposed on an influenza DI RNA delivery vehicle for clinical use is pre-existing immunity to IAVs. This is tractable as there are other human strains available with properties similar to PR8 (little human immunity and the ability to bind both types of cell receptor) that could be used as alternative delivery vehicles. In addition, the respiratory tract to which the DI virus is delivered has evolved a degree of immune privilege that minimizes immune responses to otherwise harmless environmental antigens [25,26]. If this was not so, the respiratory tract would respond immunologically to a myriad of airborne materials, wasting immune resources and causing immune-mediated pathologies. However, at the same time the respiratory tract has to combat pathogenic microorganisms, and this balance between tolerance and defence is clearly imperfect as evidenced by an array of respiratory allergies where the immune system is responding unnecessarily. This suggests that the respiratory tract immune response is likely to be more tolerant of, and respond less to DI viral antigens than the same antigens injected systemically. However, the only immune response that could reduce the effectiveness of a DI virus is antibody, and this would have to be present in the external secretions in sufficient concentration to be neutralizing. Once inside a cell the DI RNA is no longer subject to adaptive immunity. As others have described [27], 244 DI RNA is transcribed *in vivo* into mRNA(s) and complementary RNA, but with no functional segment 1 there is no *de novo* polymerase and no RNA amplification. Some individual full-length viral RNAs that survive the UV irradiation to remove infectivity, will also make mRNA and cRNA, but as 244 intrinsically lacks the PB2 protein there can be no amplification of the cRNA. Thus little, if any, protein is made and the cell containing DI RNA does not invite immune attack. DI RNAs trigger innate immune responses (see below) but these do not appear to have any deleterious effect on the DI RNA.

A desirable aspect of promoting resistance to respiratory virus infections with DI virus is to be able to give repeated intranasal dosing, but the possibility of stimulating adaptive immunity that would neutralize the next dose of DI virus is a concern. Neutralizing antibody on the luminal side of the respiratory tract is the most likely immune component to antagonise the successful (re)administration of DI influenza virus. This could neutralize the DI virus and prevent the DI RNA from entering a cell. The issue of immunogenicity can be managed by sub-immunogenic dosing as long as this is consistent with adequate protection. In studies using multiple administrations of 244 DI virus, subsequent challenge with the paramyxovirus pneumonia virus of mice (PVM) showed that an immune response, which would prevent the successful uptake of DI virus and the subsequent stimulation of interferon type I, had not been generated in the respiratory tract (unpublished data).

Treatment of mice with a single dose of an immunogenic amount of 244 DI virus (12 µg virus protein) stimulates serum HA-specific neutralizing antibody that protects against a very high lethal intranasal influenza A viral challenge given some weeks later. Two such high doses were given a week apart and were followed by a third, lower dose of DI virus (1 µg) one week later. These mice were then challenged with PVM on the next day were clearly protected. This showed that the final intranasal DI virus dose had not been neutralized despite the high levels of serum antibody that are stimulated under these conditions.

### 4.1. Does DI Virus Synthesise Any Antigen?

After the uncoating process that involves fusion of the viral and endosomal membranes, influenza viral RNPs (including the DI RNP) migrate to the cell nucleus where all viral RNA synthetic events take place. It is likely that 244 DI RNP directs the synthesis of positive strand complementary (c) RNA and mRNA. The cRNA is not replicated as this would require *de novo* synthesis of viral polymerase and the DI particle lacks the full-length RNA segment 1 to encode the PB2 component. The UV irradiation to remove helper virus infectivity means that at least one full-length segment per virion will have suffered a UV hit, but the kinetics suggest that more than one segment will have been inactivated. Stochastically the larger segments are most likely to be hit. Thus, some of the seven full-length RNAs in a DI virus particle will still be intact and will synthesise cRNA and mRNAs. The cRNA cannot be replicated as no new functional polymerase can be made, but mRNAs could direct the synthesis of their cognate proteins. This could lead to expression of viral envelope proteins and/or MHC-viral peptide complexes on the cell surface. The amounts would be very low, and the long lasting persistence of DI 244 RNA in mice suggests that any protein expression must be below the level, which would make the cell a target for cytotoxic immune effectors. By contrast DI RNA from DI virus-treated and infected ferrets is cleared at the same time and the same rate as infectious virus [28]. This probably results from the action of adaptive immune effectors (particularly virus-specific cytotoxic CD8^+^ T cells [29]) attacking infected cells that are producing DI and infectious viruses.

### 4.2. Some Elements of Adaptive Immunity Are Needed for DI Virus-Mediated Protection *in Vivo*

Severe combined immunodeficient (*scid*) mutant mice produce no functional B and T cells, but have a normal complement of NK cells. Such mice were completely protected from a lethal influenza virus challenge by treatment with DI 244 virus while infected mice given control inactivated DI virus became ill and died [30]. However, a week later the mice, which had been so well protected by DI virus, developed a typical influenza-like illness and died. This suggests that a functional B- or T-cell based adaptive immune response is required in the later stages of DI virus-mediated protection, probably to clear the infection.

### 4.3. DI Viruses Stimulate Innate Immunity

There is increasing evidence that the innate immune system is stimulated by DI viruses *in vivo* [12]. Defective and DI viruses, and particularly those that possess copyback/snapback genomes that are extensively double-stranded, stimulate the production of interferon type I [31,32,33,34,35]. It is now proposed that defective genomes are key to providing danger signals that stimulate host antiviral responses and combat virus-coded antagonists of the host’s defence mechanisms [12]. However, in these studies the authors use DI paramyxoviruses, which may differ significantly from DI influenza viruses, and because the DI genomes are uncloned, unspecified and heterogeneous. Defective genomes act on dendritic cells and stimulate antiviral genes and a unique set of proinflammatory cytokines and chemokines; they promote the maturation of dendritic cells [36], which in turn promotes the activation of T cells, and T- and B-cell-mediated adaptive immunity [37]. This pathway commences with the recognition of pathogen-associate molecular patterns (PAMPS) within DI RNAs. PAMPS bind to the intracellular RNA helicases encoded by the retinoic acid-inducible gene 1 (RIG-1) and the melanoma differentiation-associated protein 5 gene (MDA5) of dendritic cells, and activate transcription factors which lead to the expression of interferon type I, and other proinflammatory factors. Upregulation of RIG-1 but not MDA5 by defective genomes is dependent on interferon type I, but upregulation of MDA5 is dependent on the stimulation of the expression of interferon response factor 3-responsive genes [38,39]. Only lung cells that contained defective genomes made interferon type 1β; those containing only standard genomes did not [40]. The power of this pathway is shown by the preferential binding of influenza DI RNAs over full-length RNAs to RIG-1 [41], and that it is ongoing in the presence of virus-encoded antagonists of host defence mechanisms [12].

Influenza DI RNA 244 is interesting as it contains no major regions of double-strandedness but after intranasal administration with DI virus stimulates production of type I interferon in the lung. This is highly significant as it suggests that 244 DI virus could protect against all interferon-sensitive non-influenza A respiratory viruses, including those grouped together as ILI (influenza-like illnesses). Data from studies with mice that lack a functional type I interferon receptor show that interferon type I is absolutely required for protection from the non-influenza A respiratory viruses pneumonia virus of mice and influenza B virus [30,42]. Other innate immune responses may also be stimulated by DI virus and contribute to protection, as disease in the DI virus-treated interferon receptor-null mice was delayed compared to that in control interferon receptor-null mice [30]. Interferon type I is not required for protection of mice from IAVs, although it may contribute to that protection [43]. *In vitro*, a strong interferon type I β response was made in MDCK cells infected with a virus stock containing a large amount of influenza DI RNAs, and this was accompanied by a high level of apoptosis induction [44]. Such findings are consistent with the preferred binding of RIG-I to small influenza A RNAs [41].

### 4.4. DI Virus Facilitates Immunization against the Wild Virus by Converting a Virulent Infection into an “Attenuated Live Vaccine”

Mice given a lethal dose of IAV, influenza B virus, or pneumonia virus of mice and treated with 244 DI virus showed no clinical disease and no weight loss, yet developed solid immunity to the infecting virus [14,43]. 244 DI virus, whether through intrinsic interference (with IAVs) or by stimulation of interferon (with non-IAVs), does not eliminate the replication of infectious virus in the lungs but self-evidently reduces it to a subclinical level [14,43]. Thus, in essence, the DI virus converts the virulent virus infection into a quasi-live attenuated vaccine. The resulting immunity is local, directed against whole virus and/or infected cells, and stimulates both B and T cell elements of the adaptive immune response. Immunity will be stimulated by the infecting virus and the DI virus, as the latter is replicated by the infecting IAV, packaged with all the proteins encoded by the challenge virus, and can represent the majority of the virus load produced. The resulting immunity is well able to withstand reinfection with a massive lethal dose of the infecting virus [14,45].

## 5. Duration of Protection by DI Influenza Virus

One of the several surprising properties arising from the study of influenza DI RNA has been its duration of residence in the cell. Inoculation of MDCK cells with a preparation of non-infectious DI virus converted these cells which are normally permissive for IAV, into a culture that was totally resistant to infection, and after several passages (involving trypsinization, cell dilution and reseeding) the cells were still refractory to infection [46]. No cellular pathology was observed and the rate of cell division was unaffected by presence of the DI RNA. Data were consistent with each cell receiving several copies of DI RNA. The DI RNA, which resides in the nucleus and is non-replicating, appeared to assort to daughter nuclei at random or else some cells would not have received any DI RNA and would have been susceptible to infection. Eventually dilution of the DI RNA by cell division and/or degradation of the DI RNA returned the cells to complete susceptibility to infectious virus.

After intranasal inoculation of mice with DI virus under light anaesthesia DI RNA is found in the lung, which is where infectious IAV multiplies. After finding that DI RNA remained in cells in culture for many weeks and conferred resistance upon them (see above), it was of interest to see how long the protective activity of the DI RNA (in the absence of infectious IAV) persisted in the lung. Determining this is complicated by the immunogenicity of the DI virus, as a large (12 µg) dose of DI virus elicits viral haemagglutinin-specific serum antibody (and possibly other immune responses), which protect mice from infection with intranasal infectious challenge IAV. Such protection has to be the result of adaptive immunity as the very large amount of challenge virus used exceeds the maximum that the DI virus can protect against when both are given at the same time [14]. As the DI RNA cannot replicate, and much of the coding potential of the full-length virion RNA segments within a DI virus particle is destroyed by UV irradiation, it was concluded that the immunogen was the inoculated DI virus. It was not possible to circumvent the immunity problem by using an antigenically unrelated challenge virus, as mice manufacture a strong cross-immunity that is effective across even subtypes of IAV.

One solution was to use a sub-immunogenic intranasal dose of DI virus (approximately 1 µg). Such mice resisted lethal challenge with intranasal IAV at 1 week, but not at 2 weeks after treatment (unpublished data), so by two weeks they had regained susceptibility. The latter also showed that no immunity had been generated and thus that the protection observed at one week after treatment resulted from the inhibitory action of the DI RNA. Natural decay was thought to account for the loss of the DI RNA. This approach did not work with higher doses of DI virus as the mice did not regain sensitivity to challenge with intranasal IAVs due to the immunogenicity of the DI virus. However, it was possible to take the protection data for 1.2 µg DI virus and impose these on the decay curve for DI RNA in the lungs of mice inoculated with 12 µg DI virus (Figure 1) [2]. Extrapolation shows the amount of DI RNA present in lungs at 1 week after treatment when the mice were still protected and at two weeks when they were no longer protected. Mice treated with 12 µg of DI virus still had the protective amount of DI RNA at five to six weeks after treatment, indicating that they would have resisted an IAV challenge for this period of time. This information is of value as the cross-immunity between IAVs found in mice is not seen in humans and thus gives an indication of the duration of clinical efficacy.

## 6. Prophylaxis and Therapy by DI Viruses

At the present time the only DI viruses that have given clear evidence of clinical promise *in vivo* are the negative-sense, single-stranded RNA IAVs [2,6] and the neurotropic, positive-sense, single-stranded RNA alphavirus, Semliki Forest virus (SFV) [5]. There is a greater body of data for the DI influenza viruses than for DI SFV, but together they show that DI viruses are effective against infections of different organ systems–the respiratory tract and the central nervous system respectively. The DI SFV data spell out some important lessons. When given intranasally, highly virulent strains of SFV travel up the olfactory nerve to the brain and cause a fatal, descending encephalitis with hind limb paralysis. Two different cloned sequences of SFV DI RNA (DI 6 and DI 19) were studied: both interfered to a similar level with infection in cell culture, but only DI 19 protected mice [13]. Intranasal co-administration of SFV DI 19 totally prevented any sign of clinical disease, although infectious virus replicated at a reduced level in the brain and mice became immune to reinfection through stimulation of adaptive immunity [47]. The reason for this difference in protection was not determined, but it illustrates the important point that not all DI sequences can protect *in vivo*, even if they interfere strongly *in vitro*.

Broadly speaking 244 DI IAV has two different mechanisms of protection. Extrapolating from the *in vitro* data, DI RNA interferes with infectious IAV *in vivo* by inhibiting the replication of infectious virus and preferentially packaging the newly synthesized DI RNA into new virions in place of the cognate full-length RNA segment. In addition there is a huge (up to 10,000-fold) amplification of the amount of DI RNA present in the respiratory tract by the infecting IAV, and reduction in the amount of new infectious virus [28,30,45]. The interpretation is that in the multi-faceted virus-versus-host process that ensues when an animal is infected, DI virus tips the balance in favour of the host. As mentioned above, other host factors from the innate and adaptive immune responses are required for the animal to recover. Type I interferon is not required for prophylaxis or therapy of IAV. DI 244 virus protects against non-influenza A respiratory viruses by stimulating interferon type I, and possibly other components of the innate immune response. The general principal that DI virus tips the host-virus relationship in favour of the host appears also to hold true for non-flu A respiratory viruses.

Prophylaxis against influenza A infections is aided by the remarkable *in vivo* stability of DI RNA. (Figure 1 and see above). Prophylaxis against non-IAVs has a shorter window of efficacy lasting just a few days, during which production of type I interferon is stimulated. Interferon has varied and far-reaching effects on host metabolism and its production in response to DI virus (and other stimuli) will almost certainly be limited by host homeostatic mechanisms. The generally low infective dose that is probably found in natural infections [24] allows maximal effect for DI virus-mediated prophylaxis, whichever mechanism is operating, as high levels of influenza A or non-IAVs can overcome the protective effects of DI virus (Table 1) [23].

The limiting factor for DI virus-mediated post-infection therapy is the increasing virus load that accrues as the infecting virus multiplies. For both influenza A and non-IAV infections of mice given a lethal dose of virus the window for therapy is a few days. It was observed that therapy of influenza A infections required a higher dose of DI virus than did non-IAVs. It is not clear why this should be so. It may be that the interference and interferon stimulation mechanisms that protect against influenza and non-IAVs have different thresholds, or that their infectious doses have different numbers of particles, and/or they have different rates of multiplication. The reduction in effectiveness of treatment as the infectious load increases is not a problem unique to DI viruses, and occurs with all anti-viral and anti-microbial compounds.

## 7. Resistance of IAVs to Influenza DI RNA Is Highly Unlikely

With a mutation rate of approximately 10^−5^ nucleotide changes per round of replication, no error-correction, and a multiplication cycle of a few hours, resistance of RNA viruses to antivirals can arise rapidly. Resistance of a virus to interference by a cognate DI genome would occur if the sequence encoding the viral replicase mutated so that it no longer recognised and replicated the DI genome. However, a virus could only survive and be replicated if the replicase recognition sequence of the infectious virus itself mutated coordinately to compensate for the change in the replicase. What makes it especially unlikely that an influenza virus could escape the interfering action of its DI RNA is that the infectious genome is composed of 8 separate RNA molecules, which, together with the DI RNA, are all replicated by the same replicase enzyme complex. Thus a viable resistant virus could only arise if there was simultaneous mutation of the recognition sequences of each of the 8 full-length genome segments. The chance of this arising would be (10^−5^)^8^ or 10^−40^. In addition there would have to be at least one commensurate mutation in the RNA encoding the polymerase complex (comprising the PB1, PB2 and PA proteins) which would reduce the chance of an escape mutant to 10^−45^, or 1/1000,000,000,000,000,000,000,000,000,000,000,000,000,000,000.

The strength of this argument is exemplified by the generation of monoclonal neutralizing antibody escape mutants of IAV. This requires a coding mutation in the gene expressing the haemagglutinin protein such that neutralization is abrogated. The selection is so strong that with one monoclonal antibody a virus population becomes predominantly escape mutant in a single passage in embryonated eggs. However, when virus is incubated simultaneously with two monoclonal antibodies directed against separate and discrete epitopes, no escape mutants are found [48]. The mutation rate for a double escape mutant would be (10^−5^)^2^ or 10^−10^, which exceeds the yield of infectious virus per cell.

## 8. Are Respiratory Viruses Likely to Become Resistant to Interferon Stimulated by Influenza DI RNA?

The RNA of 244 DI virus is responsible for stimulating interferon type I since prolonged UV irradiation, sufficient to abrogate protection from influenza A challenge but not to denature virus haemagglutinin and neuraminidase activities, abrogates both interferon production and protection [43]. Proof that induction of interferon type I is essential for DI virus-mediated protection from non-flu A respiratory viruses was provided by its failure to protect transgenic mice that lack a functional interferon receptor. Interferon type I is part of the normal host response to virus infections, and viruses have evolved a variety of ways of ameliorating the antiviral effects of interferon [49,50]. The pathway by which DI RNA stimulates interferon is not known, but it probably activates the same mechanism(s) as infectious viruses. Infectious viruses routinely stimulate interferon and have already evolved mechanisms to counter its effects, so interferon made in response to DI RNA is unlikely to change the status quo. Making interferon in response to DI RNA helps the host defend itself from infection, so there is unlikely to be evolutionary pressure for the host to reduce its responsiveness to the DI RNA.

## 9. A Guide to Finding New DI Virus Candidates for *in Vivo* Treatments

Naturally generated DI virus comprises a population of DI genomes that are heterogeneous in sequence and interfering ability, and these continue to evolve as the DI virus is passaged. In order to have a stable product that can be quality controlled it is essential to have a defined DI genome that can be monitored by sequencing. This can be achieved by cloning DI genomes from a heterogeneous population and creating a range of DI viruses each with a unique DI genome sequence. Further passage can be minimized to avoid evolution of the cloned DI genome and the possibility of other DI genomes arising *de novo*. Ideally the DI virus should be generated in the target tissue or cells derived therefrom, as the cell is known to influence both the generation and propagation of DI RNAs [6]. These cloned DI viruses are then tested to determine which are the most effective at interference or protection. Such tests should be carried out in the desired target cell, tissue or animal host, as active interference in cultured cells is not necessarily indicative of protection *in vivo* [13]. The creation of artificial “DI genomes” by deletion of sequences from an infectious genome is not an effective approach as no such molecule is reported to be interfering [51,52,53,54]. There appear to be subtleties of sequence and/or structure of a DI genome, which are not understood at the present time but are necessary for its successful functioning. It is better to let nature select viable DI sequences, which can then be characterized and assessed for appropriate protective capacity.

All DI viruses are replicated *in trans* by a coinfecting helper virus, and ideally this should be removed or inactivated before treating cells or animals, as large amounts of infectious virus can still be present. However, infectious virus is usually a relatively minor component of a DI virus preparation (<1%) and adds little to the total virus mass present. DI and infectious viruses are antigenically identical. Usually DI and infectious viruses cannot be physically separated and even when this can be done (as in the case of DI rhabdoviruses) it is not an efficient process [8]. As discussed, helper infectivity is removed by UV irradiation.

Protection that involves amplification of the DI genome by a closely related virus can only occur if the genomes of DI and infectious viruses enter the same cell. This is facilitated if they are inoculated in close physical proximity and both use the same cell receptors. This is less relevant if the DI virus acts by stimulating interferon or other mediators of innate immunity, as these can influence cells regardless of virus receptor specificity. However, the amount of interferon stimulated by DI influenza virus is relatively low [43], so again protection by this mechanism is optimized if the DI virus is administered to a site close to where the infecting virus is expected to first enter the animal.

A relatively large amount of DI virus may be needed to protect an animal, as it is necessary for a significant proportion of cells of the target tissue to receive at least one DI genome. In this way, under conditions of prophylaxis, there is a good chance of the DI and infectious virus genomes coming together in the same cell and interacting so that infectious virus can amplify the DI genome before the infectious virus can replicate unimpeded. The number of DI and infectious genomes for successful protection depend on many variables including the amount of DI virus inoculated, the number of susceptible cells, the dose of infecting virus, and the reproductive rate (*R_0_*) of both the DI genome and the infecting virus. (*R_0_* is the average number of secondary infections (infected cells) produced by one infected cell when all individual cells are susceptible). Thus to have some chance of success, a ballpark figure for starting an initial titration would be , say, 10^9^ to 10^10^ particles of DI virus (approximately 1 µg of DI virus protein). Establishing an effective dose is an empirical process.

## 10. Potential Clinical Applications of DI Viruses

There is no disputing that new antivirals are badly needed, and the more broad-spectrum their activity the better. While DI viruses have been touted as antivirals for the clinic for many decades, success has proved elusive even in preclinical tests (but as discussed above there is now a level of understanding which should improve the discovery process to identify candidates for further development). The example of influenza 244 DI virus is an exciting and promising start and, while no DI virus has yet entered a clinical trial, consideration of how this might come about will inform the route to the clinic for any DI virus candidate.

Clinical testing of a DI virus requires convincing and extensive preclinical data. DI virus occupies a unique position in the antiviral lexicon. It is clearly a biological product, not a conventional small molecule antiviral. While a DI virus looks superficially like a vaccine it is clearly very different in its mode of action and therefore in its clinical profile. In particular the aim with a DI virus is to use it at a sub-immunogenic level and avoid any immunity that obviates repeat treatments, so that repeated dosing can be given as required. In addition to this new paradigm can be added consideration of whether or not a DI virus is a live organism: it cannot reproduce itself although in the case of DI 244 it has limited synthetic activity and can transcribe, but not replicate, its RNA. Left on its own in a cell or in the presence of non-IAVs the DI RNA decays, but with an infectious IAV genome in the same cell, the DI RNA is amplified enormously. However, progeny DI virus has the same antigenicity as the infectious virus, and is cleared at the same time as infectious virus at the end of the infection [45].

As will be evident from the foregoing discussion there is no “one size fits all” answer for the clinical appraisal of DI viruses. Each disease presents a unique clinical scenario that offers an array of challenges when considering treatment by DI virus. The upside is that there is no shortage of potential candidates, as many, if not most, viruses make DI genomes and DI viruses, although the situation is not fully clear for retroviruses. The ease with which a virus spawns DI genomes/viruses varies hugely [55,56,57]. Few DI genomes have been cloned, and most reports of DI virus activity *in vivo* have been based on mixed populations of DI genomes, and cannot be relied on as they represent an average of the effects of several different DI molecules. There is a particular dearth of data on viruses that cause systemic infections, although vesicular stomatitis virus was investigated *in vivo* many years ago [2,5]. This topic needs revisiting in view of current knowledge. Apart from the examples of influenza A and Semliki Forest virus discussed above, sound preclinical data will be required before the clinical potential of other DI viruses can be assessed.

Once a disease is selected for investigation and treatment, the relevant DI genomes have been cloned, and at least one of them shown to have good preclinical efficacy, then the matter of administration has to be decided. Treatment of respiratory tract infections, such as influenza, by DI virus, as discussed above, can be can be carried out intranasally or orally by droplet spray or aerosol administration. However, there appears to be compartmentation of the protective effects of DI virus, as introduction of DI influenza virus into the respiratory tract does not in inhibit viruses that infect systemically. For example, interferon type I stimulated by influenza DI 244 in the respiratory tract was active locally in the respiratory tract, but not against a systemic infection with encephalomyocarditis virus (unpublished data). Presumably the small amount of interferon made locally in the respiratory tract is insufficient to be effective systemically.

The majority of known infections affect the respiratory tract, the gut, the urogenital system, or systemically. Viruses can gain entry to the body directly via the respiratory tract; oral intake of virus can lead to respiratory tract or gut infections, and the urogenital system is infected directly. Systemic infections can start in the respiratory tract (e.g., measles [58], SFV [59]), orally (e.g., polio viruses [60]) or by the skin barrier being broken by injection of viruses by infected arthropods (e.g., yellow fever virus [61]) or virus-contaminated syringes (e.g., hepatitis B virus [62], human immunodeficiency virus type 1 [63]). The respiratory tract can be treated with DI viruses by both the intranasal or oral routes, and the gut by the oral route; orally there needs to be provision to prevent the low pH inactivation of any acid-sensitive viruses. Systemic infections that start in the respiratory tract, such as SFV, can be prevented by prophylactic treatment of the respiratory tract, but post-infection therapy is less likely to be effective when the bulk of the virus has established a systemic infection [5]. It is not clear how DI viruses would be administered systemically without some invasive procedure. This might be acceptable for a life-threatening infection but may not be tolerated for less serious diseases. The efficacy of DI virus administered systemically depends very much on how specifically it targets a particular organ or tissue. Otherwise the DI virus could become greatly diluted and ineffective.

Clinical trials to assess the efficacy of antivirals are expensive, costing many millions of dollars depending on size, complexity and duration. The simplest and most direct validation of the clinical potential of DI virus is the challenge trial in which a small group of susceptible individuals is directly infected with the target virus. These are provided by commercial companies and other agencies. The optimum deployment for any DI virus treatment is prophylaxis, as this allows the maximum ratio of DI particles: infectious virus particles and favours inhibition of the infectious virus. The duration of prophylaxis depends on the half-life of the DI genome in the cell, and this would have to be determined individually for each DI genome. The dose of infectious virus is important, as already indicated, and clinical trials (e.g., for influenza viruses) frequently use 10^4^–10^5^ infectious units per dose by intranasal instillation (e.g., [64]) to ensure infection of the test cohort; this is several orders of magnitude greatest than the aerosol dose (0.6–3 infectious units per 50% dose) which is believed to reflect the dose in nature [24]. The greater amount is needed to cause clinical signs and symptoms in the majority of recipients. Prophylactic treatment with DI viruses for many virus infections is unlikely to be achieved and a therapeutic approach will be necessary. DI viruses also have clear therapeutic benefit although, as with any antiviral, this diminishes as the virus load increases. The length of the therapeutic window depends on the rate of multiplication of the virus and the amount of DI virus that can be safely administered, and has to be determined experimentally. Thus timing of treatment is vital for success.

## References

[1] Von Magnus P. (1954). Incomplete forms of influenza virus. Adv. Virus Res..

[2] Dimmock N.J., Easton A.J. (2014). Defective interfering influenza virus RNAs: Time to re-evaluate their clinical potential as broad spectrum antivirals?. J. Virol..

[3] Nayak D.P. (1980). Influenza virus defective interfering particles. Ann. Rev. Microbiol..

[4] Nayak D.P., Chambers T.M., Akkina R.K. (1985). Defective-interfering (DI) RNAs of influenza viruses: Origin, structure, expression and interference. Curr. Top. Microbiol. Immunol..

[5] Barrett A.D.T., Dimmock N.J. (1986). Defective interfering viruses and infections of animals. Curr. Top. Microbiol. Immunol..

[6] Dimmock N.J., Myint S., Taylor-Robinson D. (1996). Antiviral activity of defective interfering influenza virus *in vivo*. Viral and Other Infections of the Respiratory Tract.

[7] Wright P.F., Neumann G., Yawaoka Y., Knipe D., Howley P.M. (2013). Orthomyxoviruses. Field’s Virology.

[8] Huang A.S. (1973). Defective interfering viruses. Ann. Rev. Microbiol..

[9] Jennings P.A., Finch J.T., Winter G., Robertson J.S. (1983). Does the higher order of the influenza virus ribonucleoprotein guide sequence rearrangements in influenza viral RNA. Cell.

[10] Duhaut S.D., Dimmock N.J. (1998). Heterologous protection against a lethal human H1N1 influenza virus infection of mice by a H3N8 equine defective interfering virus: Comparison of defective RNA sequences isolated from the DI inoculum and mouse lung. Virology.

[11] Saira K., Lin X., DePasse J.V., Halpin R., Twaddle A., Stockwell T., Angus B., Cozzi-Lepri A., Delfino M., Dugan V. (2013). Sequence analysis of *in vivo* defective interfering-like RNA of influenza A H1N1 pandemic virus. J. Virol..

[12] López C.B. (2014). Defective viral genomes: Critical danger signals of viral infections. J. Virol..

[13] Thomson M., White C.L., Dimmock N.J. (1998). The genomic sequence of defective interfering Semliki Forest virus (SFV) determines its ability to be replicated in mouse brain and to protect against a lethal SFV infection *in vivo*. Virology.

[14] Dimmock N.J., Rainsford E.W., Scott P.D., Marriott A.C. (2008). Influenza virus protecting RNA: An effective prophylactic and therapeutic antiviral. J. Virol..

[15] Lamb R.A., Krug R.M., Fields B.N., Knipe D.M., Howley P.M. (1996). *Orthomyxoviridae*: The viruses and their replication. Fields Virology.

[16] Guilligay D., Tarendeau F., Resa-Infante P., Coloma R., Crepin T., Sehr R., Lewis J., Ruigrok R., Ortin J., Hart D.J. (2008). The structural basis for cap binding by influenza virus polymerase subunit PB2. Nat. Struct. Mol. Biol..

[17] Baron J., Baron M. (2013). Creation of a completely helper cell-dependent recombinant morbillivirus. J. Gen. Virol..

[18] Noda T., Kawaoka Y. (2010). Structure of influenza virus ribonucleoprotein complexes and their packaging into virions. Rev. Med. Virol..

[19] Hutchinson E.C., von Kirchbach J., Gog J., Digard P. (2010). Genome packaging in influenza A virus. J. Gen. Virol..

[20] Scholtissek C., Stech J., Krauss S., Webster R.G. (2002). Cooperation between the hemagglutinin of avian viruses and the matrix protein of human influenza A viruses. J. Virol..

[21] Beare A.S., Schild G.C., Craig J.W. (1975). Trials in man with live recombinants made from A/PR/8/34 (H0N1) and wild H3N2 influenza viruses. Lancet.

[22] Meng B., Marriott A.C., Dimmock N.J. (2010). The receptor preferences of influenza viruses. Influenza Respir. Viruses.

[23] Dimmock N.J., Marriott A.C. (2006). *In vivo* antiviral activity: Defective interfering virus protects better against virulent *Influenza A virus* than avirulent virus. J. Gen. Virol..

[24] Tellier R. (2006). Review of aerosol transmission of influenza A virus. Emerg. Infect. Dis..

[25] Umetsu D.T., Dekruyff R.H. (2004). Regulation of tolerance in the respiratory tract: TIM-1, hygiene, and the environment. Ann. N. Y. Acad. Sci..

[26] Hoyne G.F., Tan K., Corsin-Jimenez M., Wahl K., Stewart M., Howie S.E., Lamb J.R. (2000). Immunological tolerance to inhaled antigen. Am. J. Respir. Crit. Care Med..

[27] Akkina R.K., Chambers T.M., Nayak D.P. (1984). Expression of defective-interfering influenza virus specific transcripts and polypeptides in infected cells. J. Virol..

[28] Dimmock N.J., Dove B.K., Meng B., Scott P.D., Taylor I., Cheung L., Hallis B., Marriott A.C., Carroll M.W., Easton A.J. (2012). Comparison of the protection of ferrets against pandemic 2009 influenza A virus (H1N1) by 244 DI virus and oseltamivir. Antivir. Res..

[29] Stambas J., Guillonneau C., Kedzierska K., Mintern J.D., Doherty P.C., la Gruta N.L. (2008). Killer T cells in influenza. Pharmacol. Ther..

[30] Scott P.D., Meng B., Marriott A.C., Easton A.J., Dimmock N.J. (2011). Defective interfering influenza virus confers only short-lived protection against influenza virus disease: Evidence for a role for adaptive immunity in DI virus-mediated protection *in vivo*. Vaccine.

[31] Strahle L., Garcin D., Kolakofsky D. (2006). Sendai defective-interfering genomes and the activation of interferon-beta. Virology.

[32] Killip M.J., Young D.F., Gatherer D., Ross C.S., Short J.A.L., Davison A.J., Goodbourn S., Randall R.E. (2013). Deep sequencing analysis of defective genomes of parainfluenza virus 5 and their role in interferon induction. J. Virol..

[33] Marcus P.I., Sekellick M.J. (1977). Defective interfering particles with covalently linked +RNA induce interferon. Nature.

[34] Sekellick M.J., Marcus P.I. (1982). Interferon induction by viruses. VIII Vesicular stomatitis virus: (^+/−^) DI-011 particles induce interferon in the absence of standard virions. Virology.

[35] Marcus P.I., Gaccione C. (1989). Interferon induction by viruses. XIX Vesicular stomatitis virus-New Jersey: High multiplicity passages generate interferon-inducing, defective-interfering particles. Virology.

[36] Yount J.S., Kraus T.A., Horvath C.M., Moran T.M., Lopez C.B. (2006). A novel role for viral-defective interfering particles in enhancing dendritic cell maturation. J. Immunol..

[37] Lopez C.B., Yount J.S., Hermesh T., Moran T.M. (2006). Sendai virus infection induces efficient adaptive immunity independently of type I interferons. J. Virol..

[38] Yount J.S., Moran T.M., Lopez C.B. (2007). Cytokine-independent upregulation of MDA5 in viral infection. J. Virol..

[39] Yount J.S., Gitlin L., Moran T.M., Lopez C.B. (2008). MDA5 participates in the detection of paramyxovirus infection and is essential for early activation of dendritic cells in response to Sendai virus defective interfering particles. J. Immunol..

[40] Tapia K., Kim W.-K., Sun Y., Mercado-López X., Dunay E., Megan W., Michael A., Lopez C.B. (2013). Defective viral genomes arising *in vivo* provide critical danger signals for the triggering of lung antiviral immunity. PLoS Pathog..

[41] Baum A., Sachidanandam R., García-Sastre A. (2010). Preference of RIG-I for short viral RNA molecules in infected cells revealed by next-generation sequencing. Proc. Natl. Acad. Sci. USA.

[42] Scott P.D., Meng B., Marriott A.C., Easton A.J., Dimmock N.J. (2011). DI influenza A virus protects *in vivo* against disease caused by a heterologous influenza B virus. J. Gen. Virol..

[43] Easton A.J., Scott P.D., Edworthy N.L., Meng B., Marriott A.C., Dimmock N.J. (2011). A novel broad-spectrum treatment for respiratory virus infections: Influenza-based defective interfering virus provides protection against pneumovirus infection *in vivo*. Vaccine.

[44] Frensing T., Pflugmacher A., Bachmann M., Peschel B., Reichl U. (2014). Impact of defective interfering particles on virus replication and antiviral host response in cell culture-based influenza vaccine production. Appl. Microbiol. Biotechnol..

[45] Dimmock N.J., Dove B.K., Scott P.D., Meng B., Taylor I., Cheung L., Hallis B., Marriott A.C., Carroll M., Easton A.J. (2012). Cloned DI influenza virus protects ferrets from pandemic 2009 influenza A virus (A/Cal/04/09, H1N1) and allows protective immunity to A/Cal/04/09 to be established. PLoS ONE.

[46] Cane C., McLain L., Dimmock N.J. (1987). Intracellular stability of the interfering activity of a defective interfering influenza virus in the absence of virus multiplication. Virology.

[47] Thomson M., Dimmock N.J. (1994). Common sequence elements in structurally unrelated genomes of defective interfering Semliki Forest virus. Virology.

[48] Lambkin R., McLain L., Jones S.E., Aldridge S.L., Dimmock N.J. (1994). Neutralization escape mutants of type A influenza virus are readily selected by antiserum from mice immunized with whole virus: A possible mechanism for antigenic drift. J. Gen. Virol..

[49] Randall R.E., Goodbourn S. (2008). Interferons and viruses: An interplay between induction, signalling, antiviral responses and countermeasures. J. Gen. Virol..

[50] Weber F., Kochs G., Haller O. (2004). Inverse interference: How viruses fight the interferon system. Viral Immunol..

[51] Pattnaik A.K., Ball L.A., LeGrone A.W., Wertz G.W. (1992). Infectious defective interfering particles of VSV from transcripts of a cDNA clone. Cell.

[52] Park K.H., Huang T., Correia F.F., Krystal M. (1991). Rescue of a foreign gene by Sendai virus. Proc. Natl. Acad. Sci. USA.

[53] Collins P.L., Mink M.A., Stec D.S. (1991). Rescue of synthetic analogs of respiratory syncytial virus genomic RNA and effect of truncations and mutations on the expression of a foreign reporter gene. Proc. Natl. Acad. Sci. USA.

[54] Sidhu M.S., Chan J., Kaelin K., Spielhofer P., Radecke F., Schneider H., Masurekar M., Dowling P.C., Billeter M.A., Udem S.A. (1995). Rescue of synthetic measles virus minireplicons: Measles genomic termini direct efficient expression and propagation of a reporter gene. Virology.

[55] Fazekas de St Groth S., Graham D.M. (1954). The production of incomplete influenza virus particles among influenza strains. Experiments in eggs. Br. J. Exp. Pathol..

[56] Meier-Ewert H., Dimmock N.J. (1970). The role of the neuraminidase of the infecting virus in the generation of noninfectious (von Magnus) interfering virus. Virology.

[57] Cole C.N. (1975). Defective interfering (DI) particles of poliovirus. Prog. Med. Virol..

[58] Ter Meulen V., Billeter M.A. (1995). Measles virus. Curr. Top. Microbiol. Immunol..

[59] Atkins G.J., Sheahan B.J., Dimmock N.J. (1985). Semliki Forest virus infection of mice: A model for genetic and molecular analysis of viral pathogenicity. J. Gen. Virol..

[60] Minor P.D., Kew O.M., Schild G.C. (1983). Poliomyelitis: Epidemiology, molecular biology and immunology. Nature.

[61] Mahy B.W.J. (1999). Impact of viral diseases on the developing world. Adv. Virus Res..

[62] Mims C.A., Nash A., Stephen J. (2000). Mims’ Pathogenesis of Infectious Disease.

[63] Levy J.A. (2007). HIV and the Pathogenesis of AIDS.

[64] Killingley B., Enstone J.E., Greatorex J., Gilbert A.S., Lambkin-Williams R., Cauchemez S., Katz J.M., Booy R., Hayward A., Oxford J. (2012). Use of a human influenza challenge model to assess person-to-person transmission: Proof-of-concept study. J. Infect. Dis..

